# Protocol for *in vivo* two-photon calcium imaging of the *Drosophila* brain

**DOI:** 10.1016/j.xpro.2025.104194

**Published:** 2025-11-08

**Authors:** Yue Tian, Ruihan Jiang, Fang Guo

**Affiliations:** 1 Department of Neurobiology, Department of Neurology of Sir Run Run Shaw Hospital and School of Brain Science and Brain Medicine, Zhejiang University School of Medicine, Hangzhou 310058, China; 2 MOE Frontier Science Center for Brain Research and Brain-Machine Integration, State Key Laboratory of Brain-Machine Intelligence, Zhejiang University, 1369 West Wenyi Road, Hangzhou 311121, China; 3 NHC and CAMS Key Laboratory of Medical Neurobiology, Zhejiang University, Hangzhou 310058, China

**Keywords:** Neuroscience, Cognitive Neuroscience, Behavior

## Abstract

Two-photon calcium imaging facilitates the real-time observation of neuronal activity. Here, we present a protocol for conducting *in vivo* two-photon calcium imaging of the *Drosophila melanogaster* brain. We describe steps for fly preparation, recording chamber construction, and preparation of the buffer solution. We then detail procedures for fly brain surgery, execution of the recording, and data analysis. This protocol enables the monitoring and assessment of neuronal responses to external stimuli and the mapping of functional connectivity coupled with optogenetics.

For complete details on the use and execution of this protocol, please refer to Jiang et al.^1^

## Highlights

• Protocol for two-photon calcium imaging in live fruit flies
• Preparation of the recording chamber and buffer solution
• Guidance for fly brain surgery before recording
• Imaging and analysis of calcium activities of target neurons

## Abstract (editor highlights)

Publisher’s note: Undertaking any experimental protocol requires adherence to local institutional guidelines for laboratory safety and ethics.

## Graphical abstract

**Figure:**
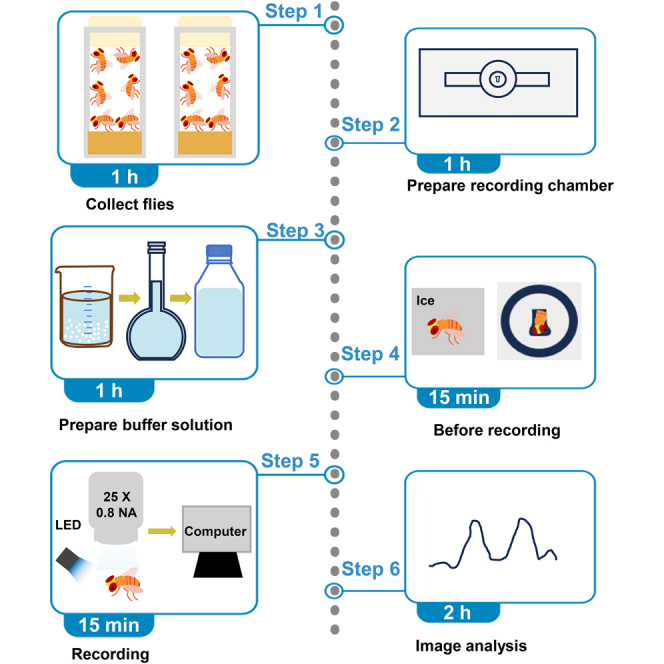

## Before you begin

Two-photon calcium imaging of live adult flies provides a more accurate reflection of physiological conditions. The flies were raised on a standard cornmeal agar medium supplemented with yeast. They were maintained at 24°C with 60% humidity under a 12-hour light/dark cycle. For optogenetics experiments coupled with two-photon calcium imaging, the flies were fed all-trans-retinal (ATR) for 3–5 days before imaging. Crosses were set up and reared under identical conditions. Both the experimental and control groups consisted of age-matched flies.

Detailed information about the flies, including genotypes and age, is provided in the key resources table in the paper.

### Innovation

This protocol presents an optimized and accessible workflow for *in vivo* two-photon calcium imaging in the *Drosophila* brain, enabling stable, high-resolution recordings of neuronal activity under physiological conditions. Compared with previous *ex vivo* or partially immobilized imaging approaches, this method improves imaging stability, reproducibility, and experimental versatility by integrating technical innovations that simplify the setup and utilize more affordable and accessible equipment.

First, we introduce a custom-designed 3D-printed recording chamber with a transparent, replaceable mobile phone screen film, allowing precise positioning and secure fixation of live flies while maintaining optical clarity and minimal mechanical stress. Optimized for both sexes, this chamber design is easily reproduced and adapted using readily available, low-cost materials, providing a cost-effective alternative to expensive commercial holders.

Second, our simplified and detailed surgical guidance minimizes tissue damage and achieves survival rates exceeding 98% after training, substantially improving success over prior reports.

Finally, we integrate open-source software tools (Fiji, MATLAB, GraphPad Prism) into a standardized analysis pipeline for motion correction, ROI selection, and quantitative assessment of calcium dynamics. This facilitates coupling with optogenetic stimulation and circadian experiments, broadening the applicability of *in vivo* imaging to diverse behavioral and physiological contexts. By relying on these freely available tools, our workflow eliminates the need for costly proprietary analysis software.

Together, these improvements make two-photon calcium imaging in *Drosophila* not only more reliable and adaptable but also significantly simpler and more accessible to a wider range of laboratories due to its reduced complexity and cost.

### Collect flies

**Timing: ∼1 h**

1. Collect the newly emerged flies and transfer them to fresh tubes.

***Note:*** Label the tubes carefully with detailed information about the flies, such as the genotype, age, and sex.

***Note:*** Collect the flies according to the purpose of the experiment.

2. Raise the flies in the incubator until recording.

***Note:*** For time-specific imaging experiments, entrain the flies for 3–5 days under 12:12 h light:dark (LD) cycles at 24°C.

**CRITICAL:** To ensure comparability, maintain identical incubation conditions and ages for both the experimental and control groups.

### Prepare the recording chamber

**Timing: ∼1 h**

**Figure 1:**
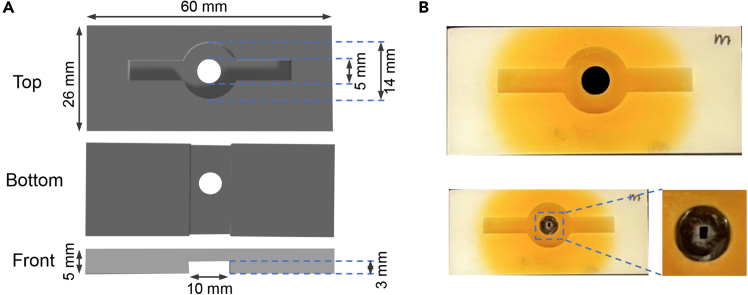
Preparation of recording chambers for two-photon live imaging (A) Scheme of the recording chambers: Length: 60 mm, Width: 26 mm, Height: 5 mm. The central part is composed of a large 14 mm diameter well, inside which a 5 mm well is centered. (B) Photograph of a recording chamber seen from above (up). The bottom view shows the recording chamber on which a mobile phone screen film has been fixed with UV glue.

***Note:*** Prepare the recording chamber before the experiment and keep it clean.

***Note:*** Chambers are reusable with proper cleaning.

***Note:*** Store at 25°C up to 3 years.

***Note:*** The approximate sizes of the hole are 1 mm with head width and 0.8 mm with abdomen width, 1.4 mm length for the female fruit fly. 0.9 mm with head width and 0.7 mm with abdomen width, 1.3 mm length for the male fruit fly.

***Note:*** The recording chamber was custom-made using resin through 3D printing, with a circular central area covered by a mobile phone screen film affixed with UV-curing adhesive. A rectangular opening was made at the center of the film using a scalpel, which was sized to accommodate the fly’s body and featured slightly widened anterior corners to accommodate the compound eyes (Figures 1A and 1B).

3. Place the recording chamber upside down.
  a. Apply a thin layer of UV adhesive around the outer rim of the central circle.
  b. Carefully position a rectangular smartphone screen protector over the glued area.
  c. Cure the adhesive by exposing it to UV light for 30 s.
  d. Use a scalpel to cut a rectangular opening half the size of the body in the center of the film.

**CRITICAL:** The dimensions in the top and bottom views are flexible; however, the 3 mm thickness of the slot in the front view should be maintained, as this space is optimized to accommodate the fly’s body and allow for natural movement.

**CRITICAL:** After imaging, immediately remove the fruit fly from the recording chamber and completely drain the recording solution. Thoroughly wipe the remaining solution in the recording chamber with lens paper.

### Prepare the buffer solution

**Timing: ∼1 h**

4. Begin by adding 450 mL of deionized, filtered water that has been treated using the Milli-Q system to a 1-L beaker.
5. Accurately weigh the following chemicals: 3.159 g of sodium chloride (NaCl), 0.1115 g of potassium chloride (KCl), 0.595 g of HEPES, 0.8557 g of trehalose, 1.7115 g of sucrose, 0.168 g of sodium bicarbonate (NaHCO_3_), and 0.078 g of sodium dihydrogen phosphate (NaH_2_PO_4_).
6. Add all of the weighed chemicals to the beaker. Use a magnetic stir bar to ensure complete dissolution, resulting in a clear, homogeneous solution.
7. Then, add 0.111 g of calcium chloride (CaCl_2_) and 0.390 g of magnesium chloride (MgCl_2_) to the saline solution. Stir with a magnetic stir bar until they are fully dissolved.
8. Transfer the resulting solution to a 500 mL volumetric flask. Rinse the container used for the dissolved chemicals three times with filtered water and add the rinsate to the volumetric flask.
9. Adjust the final volume of the solution to 500 mL.
10. Filter the solution through a sterile membrane filter to ensure sterility.
11. Finally, adjust the osmolarity to fall within the range of 280 to 300 osmoles per kilogram, and ensure that the pH is set to 7.3.

***Note:*** Initially, add approximately 450 mL of filtered water to the container.

***Note:*** The buffer solution can be stored at 4°C for 3 months.

**CRITICAL:** It is essential to adjust the pH to 7.3 using hydrochloric acid (HCl) or sodium hydroxide (NaOH). Additionally, verify that the osmotic pressure is maintained within the range of 280–300 Osm using trehalose or Milli-Q water (refer to problem 2 in the troubleshooting section).

## Key resources table

**Table:** 

REAGENT or RESOURCE	SOURCE	IDENTIFIER
**Chemicals, peptides, and recombinant proteins**		

Sodium chloride	Sinopharm Chemical Reagent Co., Ltd	Cat#10019318
Potassium chloride	Sinopharm Chemical Reagent Co., Ltd	Cat#10016318
HEPES	Sigma-Aldrich	Cat#V900477-100G
Trehalose	Sinopharm Chemical Reagent Co., Ltd	Cat#63012666
Sucrose	Sinopharm Chemical Reagent Co., Ltd	Cat#10021418
Sodium hydrogen carbonate	Sinopharm Chemical Reagent Co., Ltd	Cat#10018960
Sodium dihydrogen phosphate	Sinopharm Chemical Reagent Co., Ltd	Cat#20040818
Calcium chloride anhydrous	Sinopharm Chemical Reagent Co., Ltd	Cat#10005861
Magnesium chloride anhydrous	Sinopharm Chemical Reagent Co., Ltd	Cat#7786-30-3
Sodium hydroxide	Sinopharm Chemical Reagent Co., Ltd	Cat#10019719
Hydrogen chloride	Sinopharm Chemical Reagent Co., Ltd	Cat#10011018
All-trans-retinal (ATR)	Sigma-Aldrich	RRID:R2500-1G

**Experimental models: Organisms/strains**		

*SS00923 split-GAL4*	Bloomington Drosophila Stock Center	BDSC #75977
*UAS-GCaMP7s*	Bloomington Drosophila Stock Center	BDSC #79032
*norpA1*^−/−^ (female)	Bloomington Drosophila Stock Center	BDSC #9048

**Software and algorithms**		

Fiji/ImageJ	https://imagej.net/	RRID: SCR_003070
MATLAB	MathWorks	RRID: SCR_001622
Prism GraphPad	http://graphpad.com/	RRID: SCR_000306

**Other**		

Tweezer	RWD	Type: F11020-11
Scalpel	Sharpoint	Type: 72-1501
Mobile phone screen film	N/A	N/A
Blue LED	N/A	N/A
Red LED	N/A	N/A
UV glue	Loctite	Type: AA352
Microscope	Olympus	FV1200MPE
Beaker	In-house	N/A
Volumetric flask	In-house	N/A
Reagent bottle	In-house	N/A
Milli-Q system	Sartorius Lab Instruments GmbH & Co	Type: H20-MM-UV-T

## Materials and equipment

**Table:** Buffer solution

Reagent	Final concentration	Amount
NaCl	108 mM	3.159 g
KCl	5 mM	0.1115 g
HEPES	5 mM	0.595 g
Trehalose	5 mM	0.8557 g
Sucrose	10 mM	1.7115 g
NaHCO_3_	4 mM	0.168 g
NaH_2_PO4	1 mM	0.078 g
CaCl_2_	2 mM	0.111 g
MgCl_2_	8.2 mM	0.390 g
ddH_2_O	N/A	Up to 500 mL
Total	N/A	500 mL

## Step-by-step method details

### Before recording

**Timing: ∼15 min (for steps 1–3)**

**Timing: ∼10 min (for steps 4 and 5)**

The procedure involves anesthetizing the fruit fly, securing it in the recording chamber, and performing surgical interventions on the fly.

**Figure 2:**
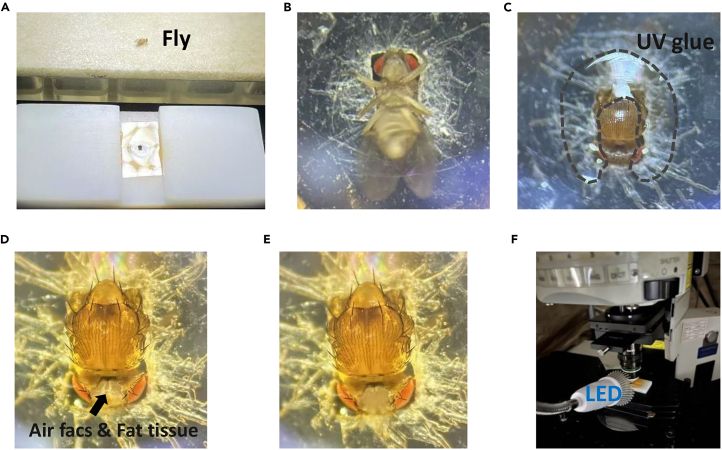
The procedures before recording (A) Fly anesthetization. (B) Transfer the anesthetized fruit fly to the inverted recording chamber. Insert head and thorax into the hole. (C) Fix the fly to the recording chamber using the UV glue. (D) Cut a rectangular patch of cuticle between the eyes of the head. (E) Remove the adipose tissue and air sacs situated above the brain. (F) Set up the experimental apparatus. Place the LED in front of the recording chamber.
2. Glue the fly to the recording chamber.
  a. Use thick-tipped forceps to transfer the anesthetized fruit fly to the inverted recording chamber.
  b. Place the fruit fly on its back in the rectangular hole in the center of the recording chamber.
  c. Carefully insert its head and thorax into the hole (Figure 2B).
    **CRITICAL:** This is a common challenge for beginners. A fly may awaken before it is properly secured in the chamber. To prolong the anesthetic effect, a cooling plate (0°C–4°C) can be placed beneath the chamber during this procedure.
    **CRITICAL:** Adjust the head and body so that they are in a straight line and not tilted. Ensure that the fruit fly is in a comfortable position.
  d. Turn the recording chamber over so that it faces upward.
  e. Apply the UV glue from one compound eye on one side and proceed around the posterior of the fruit fly to the other compound eye.
  f. Expose it to UV light for 10 s to cure the UV glue (Figure 2C).
    ***Note:*** There is no need to apply UV glue directly in front of the fruit fly’s head.
    **CRITICAL:** During this step, ensure that the fruit fly’s head remains intact and that the tweezers do not damage other body parts.
3. Perform surgery on the fly brain (Methods video S1).
  a. Introduce the solution to the central circle of the recording chamber. Ensure the fly’s head and body are completely covered by the solution.
  b. Cut a rectangular patch of cuticle between the eyes of the head (Figure 2D).
    i. Use a scalpel to make two incisions adjacent to the compound eyes near the brain.
    ii. Make a horizontal cut at the front of the fruit fly’s brain, near the ocelli.
    iii. Make another horizontal incision on the exoskeleton above the neck, at the back of the brain.
    iv. Use fine surgical tweezers to detach the rectangular patch of skin cut from the brain.
  c. Remove the adipose tissue and air sacs situated above the brain (Figure 2E).
  d. Sever the muscle no. 16,^2^ located anterior to the brain, with fine surgical forceps.
  Methods video S1. The video of the fly brain surgery, related to step 3

***Note:*** The survival rates are experience-dependent, and can reach over 98% with practice approximately 50 to achieve high proficiency.

1. Immobilize the flies aged 2∼7 days with ice (Figure 2A).

**CRITICAL:** Use the scalpel tip to delicately scratch the cuticle to prevent the excessive penetration into the brain, which could result in brain injury.

**CRITICAL:** Be careful not to puncture the brain with the tweezers when cutting the muscle.

**CRITICAL:** This is a common failure point for beginners. Because the muscles are located beneath the brain and are not directly visible, there is a risk of inserting the forceps too deeply, which can damage the brain. Repeated practice is key to avoiding this.

4. Leave the flies to recuperate for at least 5 min after surgery.
5. Assess the health of the fruit flies by observing whether they exhibit spontaneous leg movements.

**CRITICAL:** Assessing the health of the flies is also possible by looking at the neck connective and observe pulsing generated by the heartbeat. If there is no pulsing in the neck trachea, there is a good chance the fly did not survive the surgery.

**CRITICAL:** For experiments conducted at specific time points, remove the flies from the LD entrainment incubator 30 min before imaging.

**CRITICAL:** For experiments during the ZT0-12 time points, perform surgery under normal lighting conditions. For experiments during ZT12-24, extract the flies from the incubator under dark conditions. All surgical procedures were performed under dim red light to avoid circadian disruption.

### Pre-recording

**Timing: ∼5 min**

This step involves setting up the experimental apparatus and ensuring its optimal functionality.

6. Place an empty recording chamber devoid of a fruit fly under the microscope.

***Note:*** When positioning the recording chamber, ensure that the side of the fly’s head is facing the front of the operating table and is close to the experimenter.

7. Place the LED in front of the recording chamber to adequately illuminate the fly’s head (Figure 2F).

***Note:*** Use a blue LED for experiments assessing light stimulation responses, and a red LED for optogenetic coupled calcium imaging experiments.

8. Activate the LED intermittently to verify its operational status.
9. Place the fly under the microscope.

***Note:*** Only the flies in optimal condition should be placed under the microscope for subsequent imaging procedures.

### Calcium imaging recording

**Timing: ∼15 min**

These procedures are performed to acquire imaging data that aligns with the experimental goals.

10. Operate the Olympus FV1200 microscope equipped with a 25×, 0.8 NA objective lens.
11. Adjust the excitation laser to a wavelength of 920 nm.
12. Configure the imaging parameters.
  ***Note:*** The image size is 512 × 512 pixels, and the pixel resolution is 0.52 μm.
  ***Note:*** The dwell time per pixel is 2 μs, resulting in a frame rate of approximately 0.9 Hz.
  a. Set the scanning mode to either time or time and depth according to the experimental purpose.
  b. If the target area to be recorded is on the same plane, choose the time mode, that is, to image on a single plane. If the target area is located at different depths in the brain, select the time and depth mode, that is, imaging on the Z-stack.
    ***Note:*** In our study, since the target neurons project to different layers of the brain, we used the time and depth modes for z stack imaging.
    ***Note:*** The scanned depth is modified based on the morphological pattern of the recorded neurons.
    **CRITICAL:** Ensure that the parameters of the experimental groups and control groups are consistent.
13. Modify the color of LED stimulation according to the experimental requirements.

***Note:*** Use a blue LED for light response testing and a red LED for optogenetic activation. Align the frequency of LED illumination with the imaging frame rate.

**CRITICAL:** The timing of the LEDs turning on and off for each frame is meticulously controlled to prevent overexposure of the recorded neurons due to excessive illumination from the LED.

**CRITICAL:** For faster, single-plane imaging (e.g., 3–10 Hz), a software solution with triggering capabilities, such as ScanImage,^3^ is strongly recommended.

***Note:*** If the two-photon microscopy can be custom-built, the LED frequency could interleave with the image acquisition by installing the acquisition software or MATLAB scripts, so that the PMT does not pick up the LED light.

***Note:*** For details on the use and execution of this protocol, please refer to the publication.^1^^,^^4^

14. Save the data in .tiff format.

***Note:*** When naming files, it is essential to accurately include information about the recorded fruit flies, such as genotype, age, sex, and stimulus details.

***Note:*** The sample size should be sufficient to achieve statistical power. Generally speaking, each group should have no fewer than 5 individuals.

***Note:*** Maintain consistent environmental conditions, including temperature and humidity, during imaging. A stable and uniform environment is crucial for obtaining reliable results.

**CRITICAL:** For inter-group analysis: The original sentence about statistical power referred to the comparison between experimental and control groups (e.g., comparing the responses of different genotypes). For this, we specify that a sample size of at least three flies per group is typically required, given the technical nature of the experiments.

**CRITICAL:** For intra-trial analysis: To determine if a neuron shows a significant response to a stimulus within a single trial, we have adopted the reviewer's excellent suggestion. The protocol now states that a response is considered significant if its amplitude during stimulus presentation exceeds the mean of the baseline period by a defined threshold (e.g., 3 standard deviations for excitatory responses and 2 for inhibitory responses).

### Image analysis

**Timing: ∼2 h**

This step provides a brief overview of the imaging data analysis process.

**Figure 3:**
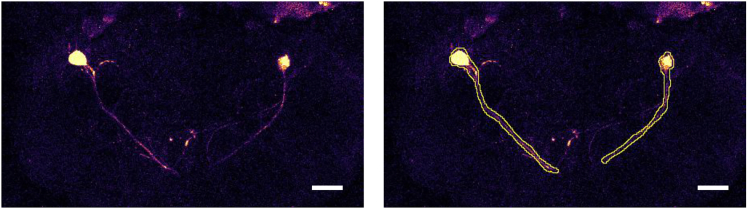
Region of interest recognition of target neurons Scale bar = 20 μm.
  d. Save the ROIs in a ZIP file.

***Note:*** Name the ROI zip file the same as the TIFF file.

15. Preprocess the recorded data using the Fiji (ImageJ) software.
  a. Import the imaging TIFF file into Fiji.
  b. Run the “Image Stacks > Z Project” command to merge the z-series stack.
  c. Define the regions of interest (ROIs) for the target neurons (Figure 3).

**CRITICAL:** Correct any motion artifacts with the TurboReg plugin in Fiji if the recorded neuron is displaced.

**Figure 4:**
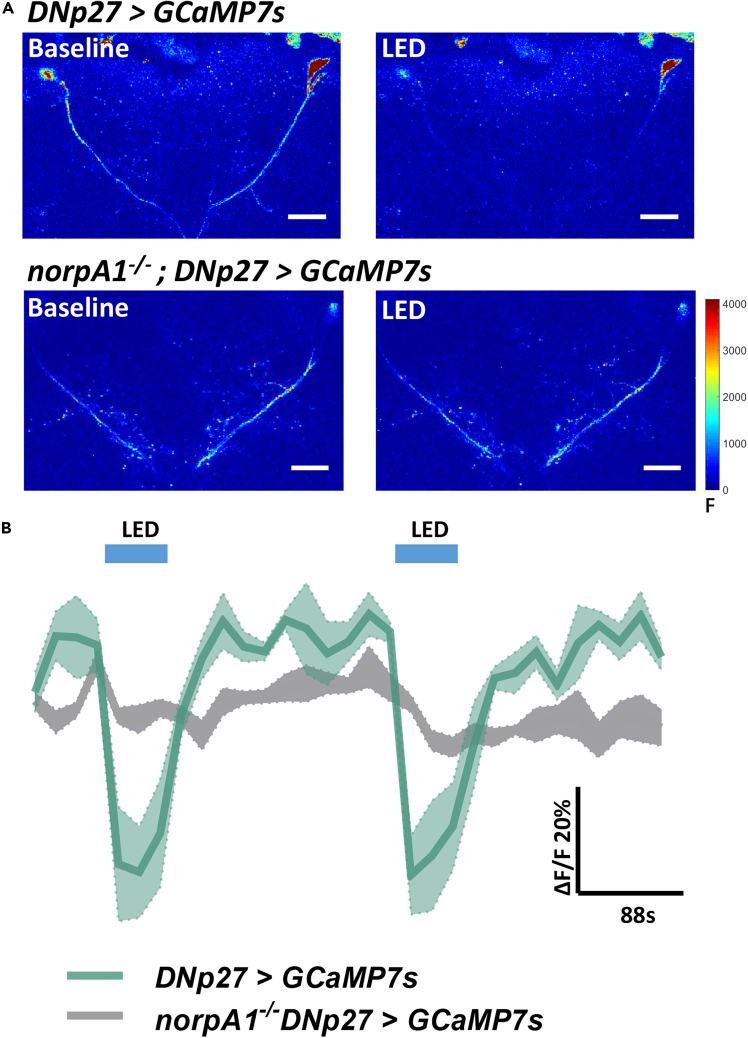
The expected outcomes of DNp27 to visual stimuli (A) Heat map of the calcium activity of DNp27 in response to a blue light pulse. (B) Trace of the recorded calcium signal of target neurons. Data are represented as mean ± SEM. Scale bar = 20 μm.

16. Analyze the GCaMP signals with MATLAB and save the data to a MAT file.
17. Plot the data and perform the appropriate statistical analyses using GraphPad Prism 8 (Figure 4).

## Expected outcomes

This protocol provides detailed instructions for calcium imaging in live fruit flies. With this technology, it is possible to observe the response characteristics of neurons to specific stimuli in real time, and also to record the spontaneous activities of neurons under specific conditions. In the research conducted by Jiang et al., the application of this technology led to the following findings that we discovered that light significantly inhibited DNp27 calcium activity during the day and at night (Figure 4).

The approach we provide here is easy to implement and inexpensive. It can record calcium signals from any brain region or neuron expressing a calcium indicator in fruit fly. It can record spontaneous calcium activity in brain regions or neurons and the neural activity responses of fruit flies to various external stimuli, including visual stimuli.^5^^,^^6^^,^^7^^,^^8^^,^^9^

## Limitations

This assay is not suitable for lines with very low expression levels of the calcium indicator. It may also impact imaging of fruit flies expressing red calcium indicators due to the indicators’ low sensitivity and tendency to bleach easily. Recording neurons in the anterior part of the fruit fly brain is difficult due to the depth of field of the two-photon microscope objective. Imaging deep brain structures may necessitate alternative approaches, such as different objectives or imaging strategies.

## Troubleshooting

### Problem 1

The solution has precipitated (related to the section on prepare the buffer solution).

Excessive storage time and improper storage conditions can both cause problems with the buffer solution. Moreover, if the chemical mixture is not fully dissolved during preparation, it can also lead to the formation of precipitates. To ensure reliable and accurate outcomes for your experiment, consider implementing the following potential strategies.

### Potential solution

• Use the solution with a storage period of less than 3 months.
• Make sure the buffer solution is stored at 4°C and not exposed to extreme conditions of heat or cold.
• Adding the drugs in the following order can help dissolve better: first, add NaCl, KCl, and HEPES. After they have completely dissolved, add trehalose and sucrose. Then add NaHCO_3_ and NaH2PO_4_ after the previous chemicals are completely dissolved. Finally, add CaCl_2_ and MgCl_2_. Use a magnetic stir bar to stir the solution throughout the process.

### Problem 2

The pH and the osmolarity of the buffer solution are not within the optimal range (Step 11 of the prepare the buffer solution section).

This is a common situation in preparing solutions. To ensure the appropriate pH and osmotic pressure, please follow the strategies below.

### Potential solution

• If the pH of the buffer solution is below 7.3, add NaOH, one drop at a time, and measure the pH again. Repeat until the pH reaches around 7.3.
• If the pH of the buffer solution is above 7.3, add HCl until the pH drops back around 7.3.
• If the osmolarity is below the optimal range, add trehalose solution and measure the osmolarity again. Repeat until the osmolarity reaches the optimal range.
• If the osmolarity is above the optimal range, add Milli-Q water until the osmolarity drops back to the optimal range.

### Problem 3

The size of the hole in the recording chamber is not appropriate for the fly (related to the section on prepare the recording chamber).

This is a common situation. To ensure the correct size, please follow the strategies below.

### Potential solution

• The body sizes of male and female fruit flies are different. Special recording chambers are made for fruit flies of different genders.
• Prepare the fruit fly before digging the hole. First, dig a small hole. Then, place the prepared fruit flies in the hole for comparison. Adjust the size of the hole according to the results of the comparison. Repeat this step until the fruit fly fits exactly into the hole.
• The head of a fruit fly is slightly wider than its body because of the compound eyes on both sides. Therefore, when digging a hole, make the opening wider at the top than at the bottom.

### Problem 4

Fruit flies are not in good condition after surgery (related to step 5 of section before recording).

There are several reasons why the fruit flies were in such poor condition. The fruit flies were not in good condition prior to the surgery. The injury to the fruit flies during the procedure, as well as the short recovery time afterwards, could both have contributed to their poor condition. To ensure reliable and accurate results, consider implementing one of the following strategies.

### Potential solution

• Do not keep fruit flies anesthetized for too long before surgery.
• During the operation, be careful not to poke the brain or body of the fruit fly with forceps or a scalpel.
• Allow the fruit flies to recover for at least 5 min after surgery.
• Avoid more than 10 sec of UV illumination.

### Problem 5

The solution seeps onto the body of the fruit fly below the recording chamber (related to step 3a of section before recording).

If the gap between the fruit fly and the recording chamber is too large, the solution will leak out. To ensure reliable and accurate results, consider implementing the following potential strategies.

### Potential solution

• Make sure there is no gap between the UV glue that fixes the fruit fly’s body and the edge of the hole in the recording chamber.
• The gap between the front of the fruit fly’s head and the hole in the recording chamber should be as small as possible (Figure 2).

### Problem 6

The brain throbs violently during imaging (related to step 3 of section before recording).

If the muscles connected to the fly’s brain start to twitch, then the brain will also throb as a result. If such a situation occurs, consider implementing the following potential strategies.

### Potential solution

• Ensure that the muscle no. 16 in front of the brain is cut.
• Fix the proboscis with UV glue to prevent regular proboscis expansion.

### Problem 7

The focal plane moves during imaging (related to step 12 of section calcium imaging recording).

The reason for this situation is the displacement of the fruit fly’s brain along the Z-axis. Implement the following potential strategies.

### Potential solution

• After finding the focal plane, allow the fruit flies to adapt for two minutes before beginning the imaging process.
• Make sure the fly brain is well-glued.
• Ensure that the muscle no. 16 in front of the brain is cut.
• Ensure the anti-vibration stage of the microscope is working properly.
• Set the microscope to focus.

### Problem 8

The calcium signal changes in the baseline (related to step 16 of section image analysis).

The possible reasons for this situation could be that the fruit flies being in poor condition, the focal plane shifting, or the environmental conditions suddenly changing. To ensure reliable and accurate results, consider implementing the following strategies.

### Potential solution

• Ensure the recorded flies are healthy and active.
• Keep the external environment stable during imaging, including temperature and humidity.
• Do not set the laser intensity too high.

## Resource availability

### Lead contact

Further information and requests for resources and reagents should be directed to and will be fulfilled by the lead contact, Fang Guo (gfang@zju.edu.cn).

### Technical contact

Technical questions on executing this protocol should be directed to and will be answered by the technical contact, Yue Tian (tianyue@zju.edu.cn).

### Materials availability

This study did not generate any new unique reagents.

### Data and code availability

The published article includes all datasets generated or analyzed during this study (Jiang et al).

## Acknowledgments

This work was supported by funding from the 10.13039/501100001809National Natural Science Foundation of China (32171008 and 32471210), the Zhejiang Provincial Outstanding Youth Science Foundation (LR20C090001), the Non-profit Central Research Institute Fund of Chinese Academy of Medical Sciences (2023-PT310-01), and the 10.13039/501100012226Fundamental Research Funds for the Central Universities (2025ZFJH01-01) to F.G. We are grateful for the technical support from the Core Facilities, Zhejiang University School of Medicine.

## Author contributions

Conceptualization, methodology, investigation, and writing – review and editing, Y.T. and F.G.; funding acquisition, resources, and supervision, F.G.

## Declaration of interests

The authors declare no competing interests.

## Supplemental information

Data S1. The 3D model of the recording chamber, related to step 3

Data S2. The algorithms used to analyze the GCaMP signals with MATLAB, related to step 16

## References

[1] Jiang R., Tian Y., Yuan X., Guo F. (2025). Regulation of pre-dawn arousal in Drosophila by a pair of trissinergic descending neurons of the visual and circadian networks. Curr. Biol..

[2] Demerec M. (1950).

[3] Seelig J.D., Chiappe M.E., Lott G.K., Dutta A., Osborne J.E., Reiser M.B., Jayaraman V. (2010). Two-photon calcium imaging from head-fixed Drosophila during optomotor walking behavior. Nat. Methods.

[4] Tian Y., Li H., Ye W., Yuan X., Guo X., Guo F. (2025). Temperature-dependent modulation of light-induced circadian responses in Drosophila melanogaster. EMBO J..

[5] Li H., Li Y., Lei Z., Wang K., Guo A. (2013). Transformation of odor selectivity from projection neurons to single mushroom body neurons mapped with dual-color calcium imaging. P Natl Acad Sci USA.

[6] Li H., Li Z., Yuan X., Tian Y., Ye W., Zeng P., Li X.M., Guo F. (2024). Dynamic encoding of temperature in the central circadian circuit coordinates physiological activities. Nat. Commun..

[7] Sun L., Jiang R.H., Ye W.J., Rosbash M., Guo F. (2022). Recurrent circadian circuitry regulates central brain activity to maintain sleep. Neuron.

[8] Wang K., Gong J., Wang Q., Li H., Cheng Q., Liu Y., Zeng S., Wang Z. (2014). Parallel pathways convey olfactory information with opposite polarities in. Proc. Natl. Acad. Sci. USA.

[9] Zhou M., Chen N., Tian J., Zeng J., Zhang Y., Zhang X., Guo J., Sun J., Li Y., Guo A., Li Y. (2019). Suppression of GABAergic neurons through D2-like receptor secures efficient conditioning in Drosophila aversive olfactory learning. Proc. Natl. Acad. Sci. USA.

